# Transcriptional silencing and activation of paternal DNA during *P* *lasmodium berghei* zygotic development and transformation to oocyst

**DOI:** 10.1111/cmi.12433

**Published:** 2015-03-30

**Authors:** Chiamaka V. Ukegbu, Jee‐Sun Cho, George K. Christophides, Dina Vlachou

**Affiliations:** ^1^ Department of Life SciencesImperial College LondonLondonUK; ^2^ The Cyprus InstituteNicosiaCyprus; ^3^ Present address: Department of MicrobiologyYong Loo Lin School of MedicineNational University of SingaporeSingapore

## Abstract

The malaria parasite develops sexually in the mosquito midgut upon entry with the ingested blood meal before it can invade the midgut epithelium and embark on sporogony. Recent data have identified a number of distinct transcriptional programmes operating during this critical phase of the parasite life cycle. We aimed at characterizing the parental contribution to these transcriptional programmes and establish the genetic framework that would guide further studies of *P* *lasmodium* zygotic development and ookinete‐to‐oocyst transition. To achieve this we used *in vitro* and *in vivo* cross‐fertilization experiments of various parasite lines expressing fluorescent reporters under the control of constitutive and stage‐specific promoters. The results revealed that the zygote/ookinete stage exhibits a maternal phenotype with respect to constitutively expressed reporters, which is derived from either maternal mRNA inheritance or transcription of the maternal allele. The respective paternal alleles are silenced in the zygote/ookinete but reactivated after midgut invasion and transformation to oocyst. Transcripts specifically produced in the zygote/ookinete are synthesized *de novo* by both parental alleles. These findings highlight a putative role of epigenetic regulation of *P* *lasmodium* zygotic development and add substantially to the emerging picture of the molecular mechanisms regulating this important stage of malaria transmission.

## Introduction

Malaria is a parasitic disease affecting almost half of the world's population. WHO reported 627 000 deaths from malaria in 2012, mostly children below the age of 5 in sub‐Saharan Africa. The disease is caused by apicomplexan *Plasmodium* parasites transmitted to humans through bites of Anopheles mosquitoes. After an infective mosquito bite, haploid sporozoites are injected into the human body and travel to the liver where they invade and replicate within hepatocytes. After some days, the infected cells rupture and merozoites enter the blood stream to infect red blood cells and embark on a continuous asexual replication–release invasion cycle responsible for the disease symptoms. Eventually, some parasites escape this asexual cycle and transform to sexually dimorphic, non‐dividing gametocytes that are infective to mosquitoes upon a blood meal. Once inside the mosquito midgut, gametocytes exit the host cells and produce male and female haploid gametes that fuse to form zygotes. The diploid zygotes embark on meiosis and soon become tetraploid. Within hours, they transform into motile ookinetes that traverse the midgut cell wall and, upon arrival to the basal side, complete meiosis and transform to oocysts. Inside an oocyst, thousands of haploid sporozoites are produced over a period of 10–15 days. They are released into the hemolymph, invade the salivary glands and infect a human host during another mosquito bite.

The time required between parasite entry in the mosquito midgut and transformation to oocyst is about 22–32 h depending on the species and environmental conditions. This is one of the most decisive phases in the entire malaria transmission cycle; the vast majority of parasites are lost during this process, mostly due to robust immune reactions mounted by the mosquito host, and indeed only few parasites survive to continue the transmission cycle. Therefore, advanced understanding of the mechanisms regulating parasite development during this time could inform the design of new methods to block disease transmission.

The *Plasmodium* erythrocytic cycle in the human host is controlled by a tightly regulated cascade of transcriptional activation, whereby different subsets of genes are switched on and off as parasites transition from one stage to another (Bozdech *et al*., 2003). A similar pattern of distinct transcriptional repertoires has been recently reported for the development of the rodent malaria parasite *Plasmodium berghei* in the *Anopheles gambiae* mosquito (Akinosoglou *et al*., 2015). At least two transcriptional programmes involving differentially regulated transcripts have been shown to support the zygote/ookinete development. The first programme (henceforth TPR1) includes transcripts that are abundant during the first 24 h post‐infection (hpi) and diminish by 48 hpi. The second programme (henceforth TPR2) includes transcripts that are *de novo* produced in the zygote/ookinete.

Many of the TPR1 transcripts are produced in the female gametocyte but remain translationally repressed by the RNA helicase DOZI; they are then supplied to the zygote as maternal mRNAs where they are translated (Mair *et al*., 2006; 2010). This process is widely observed across life kingdoms whereby quiescent female germ cells store mRNAs that are translated later in the zygote (Bettegowda and Smith, 2006; Sheth *et al*., 2010). In *Plasmodium*, products of these genes are required for zygote and ookinete development and include the ookinete surface antigens P25 and P28 as well as the Apetela 2 (AP2) transcription factor AP2‐O (Mair *et al*., 2006; 2010; Yuda *et al*., 2009). Interestingly, the TPR2 programme includes several transcripts regulated by AP2‐O itself, linked to midgut invasion and the developmental transition to oocyst. Finally, a third transcriptional programme (henceforth TPR0) that also supports the developing zygote/ookinete involves genes that are constitutively expressed throughout the parasite's sexual and sporogonic development (Akinosoglou *et al*., 2015). It includes transcripts encoding proteins with housekeeping functions such as metabolism, translation and transport.

It has increasingly become evident that epigenetic regulation is an important hallmark of the parasite's development and survival strategy within the host (Voss *et al*., 2014). The identification of the aforementioned regulatory mechanisms (Mair *et al*., 2006) and transcriptional programmes (Akinosoglou *et al*., 2015), in conjunction with the general understanding that epigenetic genomic imprinting is common in zygotic development across life kingdoms, prompted us to further investigate the parental origin of the developing *Plasmodium* zygote. Our first aim was to determine the origin of proteins and phenotypes associated with the TPR0 programme, which support housekeeping function in the developing zygote/ookinete and facilitate the production of proteins from maternally inherited transcripts of the TPR1 programme. The second aim was to characterize the origin of proteins and phenotypes associated with the TPR2 programme in order to shed light into whether both the paternal and maternal alleles are involved in the transcriptional activation of genes in the TPR2 programme or whether allelic exclusion takes place. To address these questions we used both *in vitro* and *in vivo* cross‐fertilization assays of parasite lines expressing fluorescent reporters under the control of promoters of genes belonging to the two programmes. The results of this study can help uncover the foundations for the genetic and epigenetic dissection of the *Plasmodium* zygotic development.

## Results

### Transient allelic exclusion or silencing post‐fertilization

We used two transgenic *P. berghei* lines stably expressing mCHERRY and green fluorescent protein (GFP) under the control of the constitutive elongation factor 1 subunit alpha gene promoter (*ef1α_p_)*, designated as *wt_red_230p_* and *wt_green_230p_* respectively. Transcripts of the *ef1α* gene are abundant throughout *P. berghei* development in the vector, falling within the TPR0 transcriptional programme (Akinosoglou *et al*., 2015). Constitutive GFP expression in *P. berghei* mosquito stages when GFP is placed under the control of the *ef1α_p_* has been previously reported (Franke‐Fayard *et al*., 2004). The *wt_red_230p_* line was newly generated as described in the Experimental procedures section (Fig. S1A,D), while generation of the *wt_green_230p_* line is reported in Janse *et al*. (2006). In both lines, the transgenic cassettes were integrated into the *230p* genomic locus. The morphology and growth of asexual and sexual stages of the *wt_red_230p_* line including blood stages, round and retort form zygotes, ookinetes, oocysts and sporozoites, as well as transmission to mice, were comparable to those of *wt_green_230p_* parasites (data not shown).

**Figure 1:**
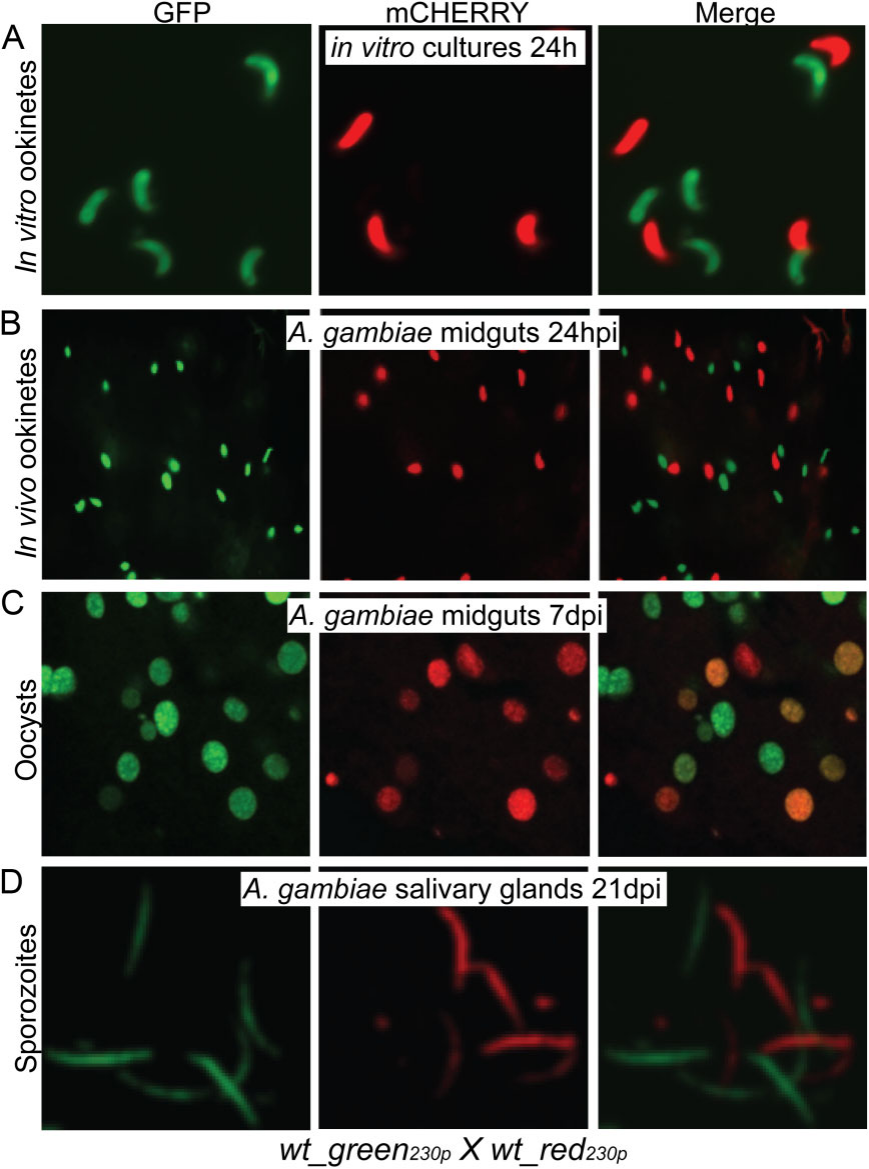
Imaging of *wt_green_230p_* and *wt_red_230p_* parasite lines derived from cross‐fertilization assays. A. Purified ookinetes from *in vitro* cross‐fertilization assays. B. Ookinetes crossing the mosquito midgut epithelium 24 hpi. C. Confocal microscopy sections of 7‐day‐old oocysts on the basal side of *A* *. gambiae* midgut epithelium. D. Confocal microscopy sections of sporozoites from homogenized *A* *. gambiae* salivary glands 21 dpi. Images were taken at ×63 magnification for the purified *in vitro* ookinetes and at ×40 magnification for mosquito tissues.

We examined the expression of fluorescent reporters in *in vitro* cross‐fertilization assays after mixing *wt_red_230p_* and *wt_green_230p_* gametocytes. Throughout the zygote and ookinete development, we observed parasites expressing only GFP or mCHERRY; GFP/mCHERRY double‐positive parasites were not detected (Fig. 1A). This observation suggested either that the (diploid and tetraploid) zygotes/ookinetes were derived exclusively from self‐fertilization of the haploid gametes of the two lines, i.e. no cross‐fertilization, or that allelic reporter gene exclusion and/or silencing occurred resulting in expression of only a single‐fluorescent protein. To confirm this observation we carried out *in vivo* cross‐fertilization assays in *A. gambiae* mosquitoes directly fed on mice previously infected with equal numbers of the two transgenic parasite lines. Microscopic observations of mosquito midguts at 24 hpi showed comparable numbers of GFP and mCHERRY expressing ookinetes invading the midgut epithelium and a total absence of GFP/mCHERRY double‐positive ookinetes (Fig. 1B). Three independent biological replicates of the *in vivo* cross‐fertilization assays were performed with highly consistent results, each using a different mouse and mosquito batch.

Intriguingly, when we continued monitoring the developmental progression of these parasites throughout their sporogonic development, a large number of GFP/mCHERRY double‐positive oocysts were detected in infected midguts 7 days post‐infection (dpi) in addition to the GFP and mCHERRY single‐positive oocysts (Fig. 1C). These observations confirmed that cross‐fertilization had occurred between the *wt_red_230p_* and *wt_gfp_230p_* gametes and that the absence of GFP/mCHERRY double‐positive zygotes/ookinetes is due to allelic exclusion or gene silencing. Only single‐positive GFP and mCHERRY sporozoites were detected in salivary glands dissected at 21 dpi, as expected due to the haploid nature of this parasite stage (Fig. 1D).

**Figure 2:**
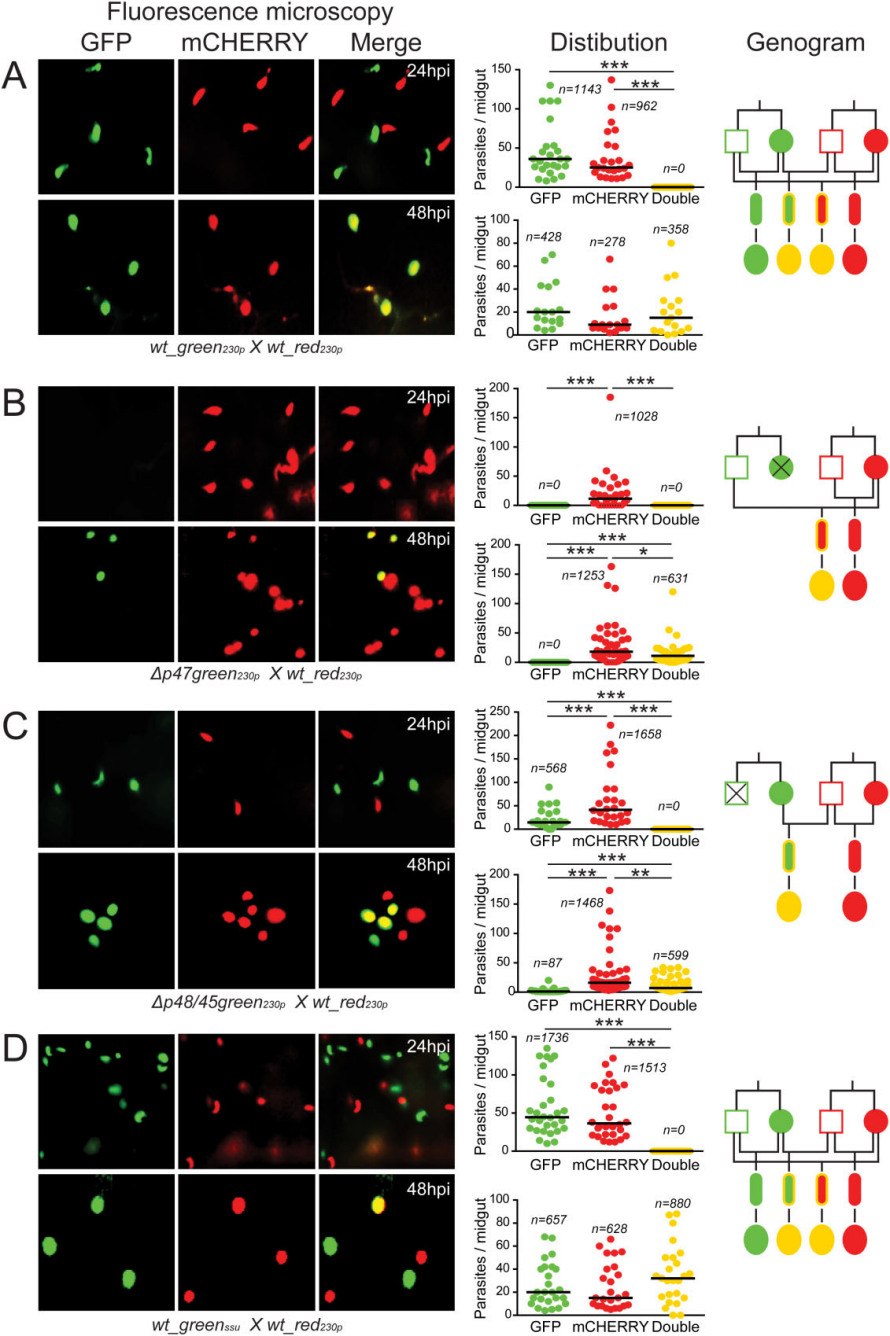
Allelic expression of fluorescent reporters placed under the control of a constitutive gene promoter. A. *wt_green_230p_* x *wt_red_230p_*. B. *Δp47green_230p_* x *wt_red_230p_*. C. *Δp48/45green_230p_* x *wt_red_230p_*. D. *wt_green_ssu_* x *wt_red_230p_*. Each part consists of three panels. The first composite panel is a collection of six representative fluorescence microscopy pictures of *A* *. gambiae* midguts fed on mice co‐infected with two transgenic parasite lines as indicated at the bottom of each part, taken at 24 and 48 hpi respectively*.* The GFP, mCHERRY and a combination of the two channels (merge) are shown. The second panel is a graph showing the distribution and median number of GFP‐positive, mCHERRY‐positive and GFP/mCHERRY double‐positive parasites per midgut at 24 and 48 hpi. The collective results from three biological replicates are shown, where *n* is the total number of parasites counted. The third panel shows a genogram summarizing the results of these cross‐fertilization experiments. Squares correspond to male gametocytes, circles correspond to female gametocytes, small ellipses correspond to ookinetes and large ellipses correspond to early stage oocysts. The colour of the outline indicates the genotype, while the fill‐in colour indicates the phenotype. Yellow outline indicates *gfp/m* *C* *herry* heterozygotes, while yellow fill‐in colour indicates GFP/mCHERRY double‐positive parasites. Black lines indicate the crosses and the resulting progeny. Horizontal black lines indicate the median parasite number. Stars indicate statistical significance determined with the Mann–Whitney *U*‐test (***P < 0.001; **P < 0.01; *P < 0.05).

We investigated the allelic exclusion/silencing phenotype in *in vivo* cross‐fertilization assays by tightly timing the mosquito midgut dissections and quantifying the number of single‐ and double‐positive parasites in each midgut. The GFP/mCHERRY double‐positive phenotype was detected in young oocysts starting at 32 hpi (Fig. 2A; Table S1) but not in any of the preceding stages including round and retort form zygotes, mature ookinetes in the blood bolus and mature ookinetes traversing the mosquito midgut. These assays were repeated three times with similar results. These data led us to hypothesize that one of the parental allelic transgenes is silenced after fertilization in the zygote/ookinete and reactivated in the young oocyst, perhaps soon after the ookinete‐to‐oocyst transition.

### Male alleles are silent in zygotes/ookinetes and reactivated in oocysts

We further investigated the putative allelic exclusion/silencing by carrying out *in vivo* cross‐fertilization assays in *A. gambiae* mosquitoes involving crossings of the *wt_red_230p_* line with the *Δp47green_230p_* line that produces only male fertile gametes (Van Dijk *et al*., 2010) and the *wt_red_230p_* line with the *Δp48/45green_230p_* (RMgm346) line that produces only female fertile gametes. Both the *Δp47green_230p_* and the *Δp48/45green_230p_* lines contain the same *ef1α_p_:gfp* transgenic cassette integrated into the *230p* genomic locus.

The *wt_red_230p_* line crossing with the *Δp47green_230p_* line resulted in only mCHERRY single‐positive ookinetes in the mosquito midgut epithelium at 24 hpi (Fig. 2B; Table S1). At 48 hpi, a comparable number of mCHERRY single‐positive and GFP/mCHERRY double‐positive oocysts were observed on the basal side of the midgut wall. These observations confirmed that the male *Δp47green_230p_* gametes cross‐fertilized with the *wt_red_230p_* female gametes and indicated that the *ef1α_p_:gfp* allele, contributed by the male genome, began to be expressed after 32 hpi in the young hybrid oocysts.

The *wt_red_230p_* line crossing with the *Δp48/45green_230p_* line resulted in ookinetes exhibiting GFP or mCHERRY single‐positive phenotypes 24 hpi (Fig. 2C; Table S1). At the oocyst stage, parasites were mCHERRY single‐positive or GFP/mCHERRY double‐positive. A small number of GFP single‐positive oocysts are thought to have had derived from a known limited self‐fertilization between gametes of the *Δp48/45green_230p_* line (Fig. S2; Table S2). These results confirmed that fertile *Δp48/45green_230p_* female gametes fertilized with *wt_red_230p_* males, but the male *ef1α_p_:mcherry* allele was not expressed before 32 hpi. Together the results from the two crossing assays indicated that the non‐active allele in the developing zygote/ookinete is contributed by the male gamete; this allele is activated again following ookinete transformation to oocyst.

To examine whether these phenotypes were specific for the *230p* genomic locus in which the fluorescent reporters had been integrated, we carried out *in vivo* cross‐fertilization assays of the *wt_red_230p_* line with the *wt_green_ssu_* line in which the *ef1α_p_:gfp* cassette was integrated into the silent *ssu‐rRNA* gene locus (529c12; Franke‐Fayard *et al*., 2004). The results revealed that the fluorescence patterns were similar to those described earlier, i.e. midgut invading ookinetes 24 hpi were either single GFP positive or mCHERRY positive whereas a large number of double‐positive oocysts were observed 48 hpi (Fig. 2D; Table S1). These data corroborated our findings that the male *ef1α_p_:gfp* allele is expressed only after 32 hpi and that our observations are independent of the genomic integration locus.

We also investigated whether our findings are specific to the *P. berghei ef1a* promoter or the *dhfr* 3′ UTR sequences, which are used in both the *ef1α_p_:gfp* and *ef1α_p_:mcherry* expression cassettes. For this, we used a transgenic line in which mCHERRY was expressed under the control of the constitutive *hsp70* gene promoter and the *hsp70* 3′ UTR (Annoura *et al*., 2014). Like the *ef1α_p_* transgenic expression cassettes, this transgene is also integrated into the *230p* genomic locus. *In vivo* cross‐fertilization assays in *A. gambiae* mosquitoes between this line, designated as *wt_hsp70_p_red_230p_*, and the *wt_green_230p_* line resulted in only single GFP‐positive or mCHERRY‐positive parasites at 24 hpi, while double‐positive oocysts began to appear only after 32 hpi (Table S1; Fig. S3). These data revealed that our findings are not limited to the *ef1a* promoter and 3′ UTR regulatory sequences.

### *D* *e novo* gene expression post‐fertilization occurs by both parental alleles

In the experiments described earlier, we used promoters of the *ef1α* and *hsp70* genes that are constitutively expressed throughout the *Plasmodium* life cycle. To investigate whether genes specifically expressed in developing zygotes are also subject to allelic exclusion/silencing, we generated two *P. berghei* transgenic lines expressing the GFP and mCHERRY fluorescent reporters under the control of the *chitinase* gene (*cht1*; PBANKA_080050) promoter (*cht_p_*). *Cht1* is expressed specifically in mature ookinetes and contributes to the penetration of the chitinaceous peritrophic matrix surrounding the blood bolus (Dessens *et al*., 2001). The two lines were designated as *wt_cht_p_green_230p_* and *wt_cht_p_red_230p_* respectively. Integration of these expression cassettes into the *230p* genomic locus was achieved by double crossover homologous recombination (Fig. S1B–C). Diagnostic polymerase chain reaction (PCR) and southern analysis on pulse field gel electrophoresis separated chromosomes (Fig. S1D) of transgenic clonal lines were used to confirm successful integration. In both lines, transcription of the transgenes began 2 h post‐fertilization (hpf) and the fluorescent reporters were detected 12 hpf onwards (Figs S4 and S5). The development of both transgenic lines was comparable with that of *wt* parasites (data not shown).

**Figure 3:**
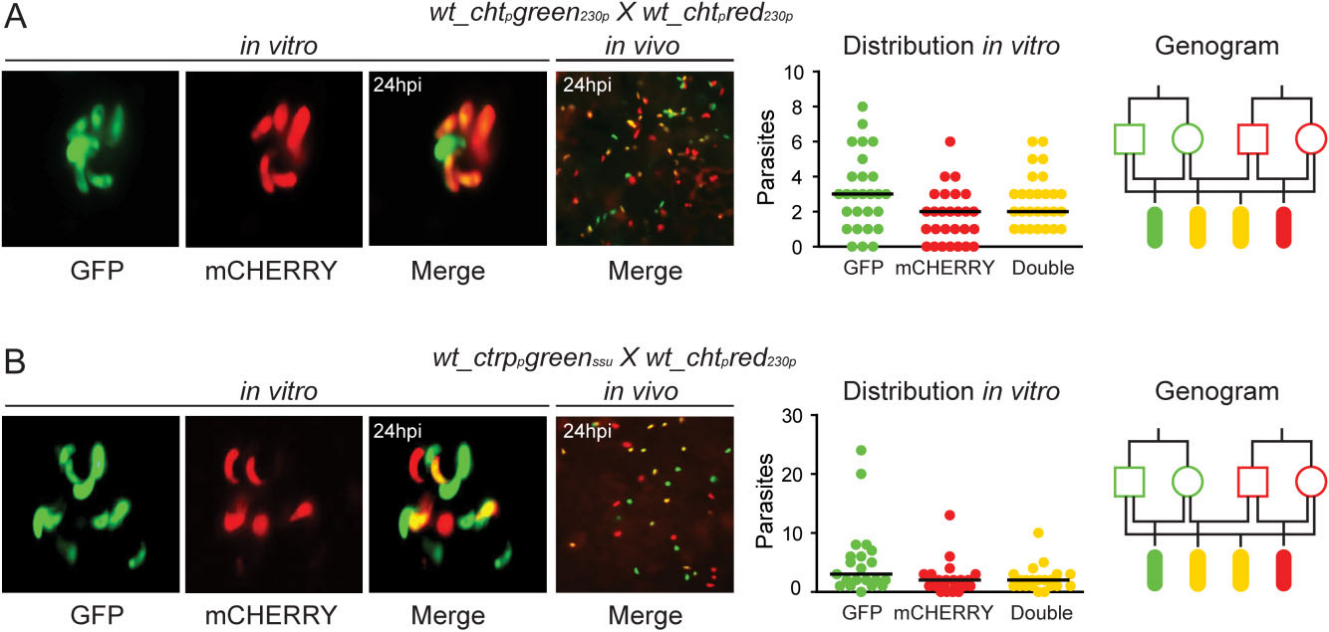
Allelic expression of fluorescent reporters placed under the control of zygote/ookinete specific promoters. A. *wt_cht_p_green_230p_* x *wt_cht_p_red_230p_*. B. *wt_ctrp_p_green_ssu_* x *wt_cht_p_red_230p_*. Each part consists of four panels. The first composite panel is a collection of three representative fluorescence microscopy pictures of *in vitro* cultured ookinetes initiated with blood from mice co‐infected with two transgenic parasite lines as indicated at the top of each part*.* The GFP, mCHERRY and a combination of the two channels (merge) are shown. The second panel shows a representative fluorescence microscopy picture of an *A* *. gambiae* midgut fed on mice co‐infected with the two transgenic parasite lines taken at 24 hpi. The third panel is a graph showing the distribution and median number of GFP‐positive, mCHERRY‐positive and GFP/mCHERRY double‐positive ookinetes in the *in vitro* culture. The fourth panel shows a genogram summarizing the results of these crosses. Squares correspond to male gametocytes, circles correspond to female gametocytes and ellipses correspond to ookinetes. The colour of the outline indicates the genotype, and the fill‐in colour indicates the phenotype. Yellow outline indicates *gfp*/*m* *C* *herry* heterozygotes, while yellow fill‐in indicates to GFP/mCHERRY double‐positive parasites. Black lines show the crosses and the resulting progeny.

Phenotypic analysis of parasite stages in *in vitro* cross‐fertilization assays of the *wt_cht_p_green_230p_* and *wt_cht_p_red_230p_* lines revealed that all possible phenotypes are obtained at the ookinete stage: GFP and mCHERRY single‐positive and GFP/mCHERRY double‐positive ookinetes (Fig. 3A; Table S3). This fluorescent pattern could be detected starting at 12 hpf. No fluorescence could be detected in activated gametocytes, gametes and zygotes (data not shown). The same pattern of fluorescent reporter expression was observed in *in vivo* cross‐fertilization assays in *A. gambiae* (Fig. 3A; Table S4). These observations demonstrated that, when placed under the control of the *cht1* promoter, the *gfp* and *mCherry* transgenes are *de novo* transcribed in developing zygotes/ookinetes from both parental alleles.

To confirm that the earlier results are not limited to the *cht_p_*, we used the *wt_ctrp_p_green_ssu_* parasite line that expresses GFP under the control of the circumsporozoite and thrombospondin‐related adhesive protein gene promoter (*ctrp_p_*; PBANKA_041290). In this line, GFP is detected in zygotes as soon as 4 hpf (Vlachou *et al*., 2004). Phenotypic analysis of ookinetes from *in vitro* cross‐fertilization assays of *wt_ctrp_p_green_ssu_* and *wt_cht_p_red_230p_* parasites revealed an all‐encompassing fluorescent pattern 24 hpf similar to that of the *cht_p_* assays: GFP and mCHERRY single‐positive as well as GFP/mCHERRY double‐positive ookinetes (Fig. 3B; Table S3). Similar results were obtained in *in vivo* cross‐fertilization assays using the *wt_ctrp_p_green_ssu_* and *wt_cht_p_red_230p_* lines. At 24 hpi, both single‐ and double‐fluorescent ookinetes were detected in the invaded mosquito midgut epithelium (Fig. 3B; Table S4). These observations indicated that genes specifically expressed in the developing zygote/ookinete are *de novo* transcribed by both parental alleles.

## Discussion

We present data suggesting that, during *P. berghei* zygotic (including ookinete) development in the *A. gambiae* midgut, transcripts produced by constitutive promoters and encoding proteins involved in housekeeping functions are provided exclusively by maternal alleles. We reveal that the respective paternal alleles are silenced during this time but reactivated for transcription and translation after invasion of the mosquito midgut, following the ookinete transformation to oocyst. We also reveal that, at the same time, transcripts specifically found in the zygote/ookinete, encoding proteins that function in midgut invasion and the developmental transition to oocyst, are synthesized *de novo* by both parental alleles. A final group of transcripts involved in zygotic development are synthesized in the female gametocyte and supplied to the zygote during fertilization as maternal mRNAs (Mair *et al*., 2006; 2010). These data add to an emerging picture of complex and tightly regulated gene expression repertoires and deduced phenotypes in the developing *Plasmodium* zygote (Akinosoglou *et al*., 2015) and expose a sophisticated genetic framework within which further studies of this important stage of malaria transmission can be conducted.

**Figure 4:**
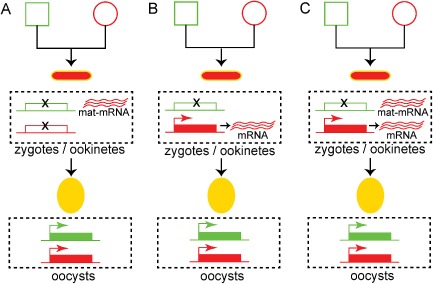
Models explaining the maternal phenotype related to the constitutively expressed reporters. A. Maternal mRNA (mat‐mRNA) contribution. Both parental alleles are silenced during zygotic development. Maternal mRNA is supplied by the female gametocyte and translated. B. Paternal allele silencing. The parental allele is silenced during zygotic development. The maternal allele is *de novo* transcribed and translated. C. Paternal allele silencing and maternal mRNA contribution. A combined model, whereby the paternal allele is silenced, while maternal mRNA is supplied by the female gametocyte and the maternal allele is *de novo* transcribed and translated. Squares correspond to male gametocytes, circles correspond to female gametocytes, small ellipses correspond to ookinetes and large ellipses correspond to early stage oocysts. The colour of the outline indicates the genotype, and the fill‐in colour indicates the phenotype. Yellow outline indicates *gfp*/*mCherry* heterozygotes, while yellow fill‐in indicates GFP/mCHERRY double‐positive parasites.

We present three hypotheses with regard to the origin of zygotic transcripts of constitutively expressed genes (TPR0 transcripts). The first hypothesis is that both the paternal and maternal alleles are silenced during zygotic development, and that transcripts are inherited to the zygote by the female gametocyte as maternal mRNAs (Fig. 4A). Indeed, a defining feature of sexual development in metazoans is the detainment of a subset of transcripts in quiescent messenger ribonucleoprotein particles in the oocytes for later translation in the zygote (Bettegowda and Smith, 2006; Sheth *et al*., 2010). These particles involve DDX6 class DEAD box RNA helicases that directly bind maternal mRNAs and are often located in structures designated as P granules (Pitt *et al*., 2000; Schisa *et al*., 2001). This hypothesis is strongly supported by the discovery of a translational repression mechanism in *P. berghei* involving the RNA helicase DOZI and additional proteins homologous to those found in P granules (Mair *et al*., 2006; 2010). This mechanism controls the expression of a large number of genes involved in zygotic development corresponding to almost half of the parasite transcriptome (Guerreiro *et al*., 2014) and including the surface proteins P25 and P28 (TPR1 programme). In conclusion, according to this first hypothesis, the maternal TPR0 phenotype of the zygote/ookinete gradually diminishes as maternal mRNAs are exhausted and is replaced by an integrated parental phenotype derived from transcriptional reactivation of both parental alleles in the young oocyst.

The second hypothesis is that, although paternal alleles are silent, maternal alleles remain transcriptionally active throughout zygotic development (Fig. 4B). This hypothesis is supported by the fact that another transcriptional programme, TPR2, is operational during the same time, starting soon after fertilization. This programme includes *de novo* synthesized transcripts encoding proteins that are exclusive to the zygote/ookinete and are involved in ookinete midgut invasion such as CHT1, WARP and CTRP. It appears to be tightly and specifically regulated by the transcription factor AP2‐O (Yuda *et al*., 2009). Therefore, it is probable that the basal transcriptional machinery remains constitutively active throughout zygotic development transcribing non‐silenced maternal alleles of the TPR0 programme, while the TPR2 programme is initiated upon availability of the AP2‐O factor, supplied as maternal mRNA by the TPR1 programme, and possibly additional transcriptional factors. Finally, a combined hypothesis is also possible, whereby both maternal and *de novo* produced mRNAs of maternal allele origin are involved in the maternal TPR0 phenotype of the developing zygote (Fig. 4C).

A conserved feature of all three hypotheses is the silencing of the paternal and perhaps the maternal alleles throughout zygotic development and their reactivation upon transformation to oocyst. This observation hints at epigenetic regulation mechanisms, pertaining to monoallelic or biallelic gene silencing respectively. In support of the paternal allele silencing concept is the finding that the paternal DNA is packaged differently in the male than the female gametocyte (Laurentino *et al*., 2011), which may explain its unavailability for transcription in the zygote/ookinete and until mitotic replication commences in the oocyst. In the last decade, it has become increasingly evident that epigenetic mechanisms are an important hallmark of the parasite's survival strategy within the host (Voss *et al*., 2014). Genes involved in host–parasite interactions, coding for virulence factors or ligands involved in red blood cell invasion, are epigenetically regulated (Cortés *et al*., 2007; Flueck *et al*., 2009), while genes involved in drug resistance are switched on or off epigenetically in an environment‐dependent manner (Sharma *et al*., 2013). Therefore, it is reasonable to hypothesize that processes involved in parasite survival in the hostile mosquito gut environment are also controlled at an epigenetic level, allowing parasites to adapt to their environment. This is supported by recent evidence showing that parasite virulence in mice is greatly affected by the passage through the mosquito gut (Spence *et al*., 2013), suggesting that epigenetic imprinting may occur during sexual development. Although the *P. falciparum* genome is largely euchromatic (active) and dominated by a code of histone modifications associated with euchromatic marks (Salcedo‐Amaya *et al*., 2009; Trelle *et al*., 2009; Gupta *et al*., 2013), clonally variant gene families found in heterochromatic loci are associated with the evolutionary conserved regulator of heterochromatin, HP1 (Flueck *et al*., 2009; Lopez‐Rubio *et al*., 2009). It remains to be explored whether such differential histone marking is also relevant during zygotic development.

The zygote/ookinete is the only stage in the entire life cycle at which parasites are non‐haploid. Therefore, infections of mosquitoes with non‐clonal parasite populations, e.g. parasite strains harbouring variations or carrying mutations in different genes, lead to allele heterozygosity. The resulting combinations of heterozygote alleles increase exponentially as the number of input strains increases. This hinders the design of genetic approaches to study the function of genes during these critical stages of malaria transmission when parasites are not clonal or when conducted in a high throughput manner. The data we present here offer an elegant genetic framework in which such approaches can be envisaged, whereby genes or genetic modifications can be placed on either side of the allelic exclusion ‘seesaw’ in order for them to be expressed or kept silent until a later time point respectively.

## Experimental procedures

### Ethics statement

This study was carried out in strict accordance with the United Kingdom Animals (Scientific Procedures) Act of 1986. The protocols for mosquito maintenance or infection with *P. berghei* through blood feeding on naïve or parasite‐infected mice, respectively, as well as for culturing parasites in mice were approved and carried out under the UK Home Office License PLL70/7185.

### Parasite cultivation and mosquito infections

The *P. berghei* clones constructed previously and reused in this study were the gametocyte‐producer ANKA 15cy1A, *Δp48/45green_230p_* (764acl1; RMgm346), *Δp47green_230p_* (765acl1*;*RMgm347; Van Dijk *et al*., 2010), *wt_ctrp_p_green_ssu_* (Vlachou *et al*., 2004), *wt_green_230p_* (507m6cl1; Janse *et al*., 2006), *wt_green_ssu_* (259cl2; Franke‐Fayard *et al*., 2004) and *wt_hsp70_p_red230_p_* (1804cl1; RMgm928; Annoura *et al*., 2014). Parasite handling and purification of blood stages were performed as described (Janse and Waters, 1995). Ookinete *in vitro* culturing was carried out as described (Rodrıguez *et al*., 2002). *A. gambiae* mosquitoes of the N'gousso strain were infected with *P. berghei* by direct feeding on mice using standard methods (Sinden, 1997; Sinden *et al*., 2002).

### Generation of *P* *. berghei* reporter lines

To introduce a constitutively expressed *mCherry* cassette into the *P. berghei* ANKA 15cy1A genome and generate the *wt_red_230p_* stable transgenic line, the *pmCherry_con_* vector plasmid was constructed. The plasmid pL0018 (MRA‐787, MR4) served as a backbone for this vector. The plasmid pL0018 contains two expression cassettes, one for the expression of the selectable marker *Toxoplasma gondii dhfr* (*tgdhfr*) under the control of the *P. berghei dhfr* promoter (*dhfr_p_*) and a second under the control of the *P. berghei ef1α_p_* that allows constitutive expression of any inserted downstream transgene. The *230p* cassette allows integration via double crossover homologous recombination. *mCherry* was amplified from *pmCherry* vector (Clonetech) as a BamHI fragment and cloned into the pCR®2.1‐TOPO® vector (Invitrogen). The BamHI fragment of the *GFP mutant 3* gene of plasmid pL0018 was replaced with the BamHI *mCherry* gene fragment.

For the generation of the *wt_cht_p_red_230p_* and *wt_cht_p_green_230p_* lines, the *pcht_p_mCherry* and *pcht_p_gfp* vectors, respectively, were constructed. First, a 820 bp fragment directly upstream of the *cht1* open‐reading frame was amplified from *P. berghei* genomic DNA as an AflII/BamHI fragment and cloned into the pCR 2.1‐TOPO vector (Life Technologies). To construct the *pcht_p_mCherry* vector, the plasmid *pmCherry_con_* (see earlier) served as a backbone for this vector. The *ef1α_p_* promoter of *pmCherry_con_* was replaced by the AflII/BamHI fragment of the *cht_p_* promoter. Similarly, to construct the *pcht_p_gfp* vector, the plasmid *pcht_p_mCherry* served as a backbone for this vector. The BamHI fragment of the *mCherry* gene of *pcht_p_mCherry* plasmid was replaced with the BamHI fragment of pL0018 containing the *gfp mutant 3* gene.

### Genotypic analysis of transgenic parasites

*P. berghei* genomic DNA was prepared from transfected blood stage parasite populations. White blood cells were removed by filtration over CF‐11 column (Whatman) and red blood cells were lysed by incubation for 20 min on ice in 0.17 M ammonium chloride. Genomic DNA was extracted using DNeasy kit (Qiagen) and subjected to diagnostic PCR to assess successful integration. Southern blot analysis on pulse field gel electrophoresis separated chromosomes of purified blood stage parasites was also performed. The blot was hybridized against a probe encompassing the *Pbdhfr* 3′ UTR cassette obtained by the *Hind III*/*EcoRV* digest of the pBS‐TgDHFR vector.

### Characterization of the *cht_p_* promoter

To characterize the timing of the expression of the fluorescent reporters under the control of the *cht_p_* promoter, a time course analysis was carried out. Briefly, blood from mice infected with either *wt_cht_p_green_230p_* or *wt_cht_p_red_230p_* parasite lines was used to set up an *in vitro* ookinete culture. The culture was split into several flasks and incubated at 21°C. At 2, 4, 8, 12, 16 and 24 h, a sample was taken and analysed by fluorescence microscopy. The rest of the culture was spun down, the red blood cells lysed in 0.17 M ammonium chloride and parasites pelleted for subsequent RNA extraction.

### Transcriptional profiling using RT‐PCR

Using the Trizol® reagent (Invitrogen), total RNA was isolated from purified mixed blood stages (MBS) and gametocytes and *in vitro* ookinetes of the *wt_cht_p_green_230p_* or *wt_cht_p_red_230p_* parasite lines. Gene‐specific primers (Table S5) were designed using Primer3 (v. 0.4.0) and used in RT‐PCR.

### *In vivo* and *in vitro* cross‐fertilization assays

We performed both *in vitro* and *in vivo* cross‐fertilization assays to assess the developmental profiles of transgenic lines and the expression of fluorescence reporters. Briefly, blood from mice infected with individual parasites was mixed in equal proportions and used to co‐infect new mice. For *in vitro* cross‐fertilization assays, infected blood at parasitaemia of 15–20% was acquired from these mice via heart puncture and used to setup *in vitro* ookinete cultures. At 24 hpf, mature ookinetes were analysed for fluorescence and also counted. Similarly, for *in vivo* cross‐fertilization assays, *A. gambiae* mosquitoes (N'gousso strain) were allowed to feed directly on the co‐infected mice with a parasitaemia 5–6%. Mosquito midgut tissues were dissected and parasite numbers and expression of fluorescent reporters were assessed by fluorescent microscopy at 24 and 32–48 hpi, 7 and 21 dpi.

### Imaging and enumeration of parasites

Following mosquito infection and dissection, midguts were fixed in 4% formaldehyde (v/v) (16% methanol free, ultrapure stock diluted in PBS, Polysciences Inc.) for 20 min at room temperature and washed three times for 10 min each in PBS. Fixed midguts were mounted in Vectashield® (VectorLabs) on glass slides under sealed coverslips. Oocyst numbers were counted at 7 dpi using fluorescence microscopy under ×10 magnification. Salivary gland sporozoites were observed at day 21 dpi. Parasites were visualized using either a Leica DMT fluorescence microscope or a Leica SP5 MP inverted confocal microscope, and images were taken using a Zeiss AxioCam HRc camera coupled to Zeiss Axiovision40 version 4.6.1.0 software or Leica LAS AF software (Leica Microsystems) respectively.

### Statistics

Statistical analysis of parasite loads in the mosquito midguts were performed using the Mann–Whitney *U*‐test.

## Supporting information


**Fig. S1.** Generation of *wt_red_230p_*, *wt_cht_p_red_230p_* and *wt_cht_p_green_230p_* transgenic parasites. Schematic representation of the *pmCherry_con_* (A), *pcht_p_mCherry* (B) and *pcht_p_gfp* (C) expression cassettes that are inserted into the *230p* locus via double crossover homologous recombination resulting in the loss of a 1 kb region of the native locus.
D. Diagnostic PCR of clonal parasites corroborating successful integration of the aforementioned expression cassettes.
E. Southern blot analysis of clonal parasites corroborating successful integration of the expression cassettes. The positions of the PCR primers used for the diagnostic PCR reactions are shown (P5, P6 and P7).
**Fig. S2.** Oocyst load in *A. gambiae* mosquitoes infected with the *Δp48/45green_230p_* parasite. The incomplete defective phenotype of the male gamete defective *Δp48/45green_230p_* transgenic parasite results in the escape of very few male gametes that are able to fertilize the normal *Δp48/45green_230p_* female gametes to form few ookinetes and thus the few oocysts observed in *A. gambiae* mosquitoes.
**Fig. S3.** Allelic expression of fluorescent reporters placed under the control of constitutive gene promoters. *In vivo* cross‐fertilization assays in *A. gambiae* mosquitoes directly fed on mice infected with equal numbers of the transgenic parasite lines *wt_green_230p_* (*ef1α* gene promoter) and *wt_hsp70_p_red_230p_*. The first composite panel includes representative fluorescence microscopy pictures of *A. gambiae* midguts fed on mice co‐infected with two transgenic parasite lines as indicated at the bottom of each part, taken at 24 and 48 hpi respectively. The GFP, mCHERRY and a combination of the two channels (merge) are shown. The second panel is a graph showing the distribution and median number of GFP‐positive, mCHERRY‐positive and GFP/mCHERRY double‐positive parasites per midgut at 24 and 48 hpi. The collective results from three biological replicates are shown, where *n* is the total number of parasites counted. The third panel shows a genogram summarizing the results of these cross‐fertilization experiments. Squares correspond to male gametocytes, circles correspond to female gametocytes, small ellipses correspond to ookinetes and large ellipses correspond to early stage oocysts. The colour of the outline indicates the genotype, whereas the fill‐in colour indicates the phenotype. Yellow outline indicates *gfp/mCherry* heterozygotes, whereas yellow fill‐in colour indicates GFP/mCHERRY double‐positive parasites. Black lines indicate the crosses and the resulting progeny. Horizontal black lines indicate the median parasite number. Stars indicate statistical significance determined with the Mann–Whitney *U*‐test (***P < 0.001).
**Fig. S4.** GFP expression in the *wt_cht_p_green_230p_* parasite line.
A. RT‐PCR analysis of *gfp* transcripts from mixed blood stages, activated (A) and non‐activated (nA) gametocytes, and parasites purified from *in vitro* ookinete cultures at 2, 4, 8, 12, 14, 16 and 24 h post‐fertilization. *Tubulin* transcripts served as a loading control.
B. Fluorescence microscopy analysis of *wt_cht_p_green_230p_* parasites purified from *in vitro* ookinete cultures at 2, 4, 8, 12, 14, 16 and 24 hpa. Parasites were also stained with a Cy3 conjugated α‐P28 antibody. Images were taken at ×40 magnification.
**Fig. S5.** Expression of mCHERRY in the *wt_cht_p_red_230p_* parasite line.
A. RT‐PCR analysis of *mCherry* transcripts from mixed blood stages, activated (A) and non‐activated (nA) gametocytes, and parasites purified from *in vitro* ookinete cultures at 2, 4, 8, 12, 14, 16 and 24 h post‐fertilization. *Tubulin* transcripts served as a loading control.
B. Fluorescence microscopy analysis of *wt_cht_p_red_230p_* parasites purified from *in vitro* ookinete cultures at 4, 8, 12, 14, 16 and 24 hpa. Bright field images are also shown. Images were taken at ×40 magnification.
**Table S1.** Parasite development in *A. gambiae* mosquitoes co‐infected with parasites constitutively expressing GFP and mCHERRY.
**Table S2.** Oocyst numbers in *A. gambiae* infections.
**Table S3.** *In vitro* cross‐fertilization assays of GFP and mCHERRY expressing parasites.
**Table S4.** Parasite development in *A. gambiae* mosquitoes co‐infected with parasites expressing GFP and mCHERRY during the zygote/ookinete stages.
**Table S5.** Primers for the generation of transgenic parasites and RT‐PCR.
