# Supplementary material for: Transcriptional silencing and activation of paternal DNA during P lasmodium berghei zygotic development and transformation to oocyst
Source: Cell Microbiol. 2015 Mar 30;17(8):1230–40. doi: 10.1111/cmi.12433 (PMC4678591; doi:10.1111/cmi.12433)
Supplement: Supplementary file 1 — Fig. S1. Generation of wt_red230p, wt_chtpred230p and wt_chtpgreen230p transgenic parasites. Schematic representation of the pmCherrycon (A), pchtpmCherry (B) and pchtpgfp (C) expression cassettes that are inserted into the 230p locus via double crossover homologous recombination resulting in the loss of a 1 kb region of the native locus. D. Diagnostic PCR of clonal parasites corroborating successful integration of the aforementioned expression cassettes. E. Southern blot analysis of clonal parasites corroborating successful integration of the expression cassettes. The positions of the PCR primers used for the diagnostic PCR reactions are shown (P5, P6 and P7). Fig. S2. Oocyst load in A. gambiae mosquitoes infected with the Δp48/45green230p parasite. The incomplete defective phenotype of the male gamete defective Δp48/45green230p transgenic parasite results in the escape of very few male gametes that are able to fertilize the normal Δp48/45green230p female gametes to form few ookinetes and thus the few oocysts observed in A. gambiae mosquitoes. Fig. S3. Allelic expression of fluorescent reporters placed under the control of constitutive gene promoters. In vivo cross‐fertilization assays in A. gambiae mosquitoes directly fed on mice infected with equal numbers of the transgenic parasite lines wt_green230p (ef1α gene promoter) and wt_hsp70pred230p. The first composite panel includes representative fluorescence microscopy pictures of A. gambiae midguts fed on mice co‐infected with two transgenic parasite lines as indicated at the bottom of each part, taken at 24 and 48 hpi respectively. The GFP, mCHERRY and a combination of the two channels (merge) are shown. The second panel is a graph showing the distribution and median number of GFP‐positive, mCHERRY‐positive and GFP/mCHERRY double‐positive parasites per midgut at 24 and 48 hpi. The collective results from three biological replicates are shown, where n is the total number of parasites counted. The third panel shows [file CMI-17-1230-s001.zip › CMI_12433_Supp-0007-Vlachou Table S2 R1.pdf]

Table S2. Oocyst numbers in *A. gambiae* infections**.**

Time	Parasite	Number of exp	Number of midguts	Prevalence (%)	Parasite density		Parasite Range	*P*-value
					Arithmetic mean	Median		
Day 10	*wt_green*$_{230p}$	2	60	90	39.8	34	0-149	<0.0001
	Δ*p48/45_green*$_{230p}$	2	61	13	0.24	0	0-4	

This table reports oocyst numbers derived from two biological replicates of infection of *A. gambiae* mosquitoes with the *wt_green*$_{230p}$ or the male gamete deficient transgenic parasite, Δ*p48/45_green*$_{230p}$. P-value was calculated using the Mann-Whitney *U*-test.
